# 241. Antibiotic Prescription in COVID Intensive Care Unit (ICU): Empiric Treatment vs. FilmArray Pneumonia (FAP) Panel Guided Treatment

**DOI:** 10.1093/ofid/ofac492.319

**Published:** 2022-12-15

**Authors:** Maria F Jimenez, Bayron Garcia, Karen M Ordonez, German A Moreno

**Affiliations:** Universidad Tecnologica de Pereira, Pereira, Risaralda, Colombia; Universidad Tecnologica de Pereira, Pereira, Risaralda, Colombia; Hospital Universitario San Jorge de Pereira, Pereira, Risaralda, Colombia; Universidad Tecnologica de Pereira, Pereira, Risaralda, Colombia

## Abstract

**Background:**

The empiric prescription of antibiotics in COVID 19 ICU patients is frequent due to the severity of disease and presentation of patients with septic shock. In this study we compared two approaches of antimicrobial prescription: empiric use vs. FilmArray pneumonia (FAP) panel guided treatment. We evaluated costs of intervention, clinical outcomes as development of hospital acquired infections (HAI), length of stay and mortality.

**Methods:**

Retrospective study. Patients with severe COVID-19 infection hospitalized in ICU of two institutions in Pereira were included. The prescription of antibiotic without FAP panel was defined as empiric. The prescription according to FAP panel results was defined as guided. Data analysis was performed in Epiinfo version 7.5.2.0. The study protocol was approved by the ethics committee of Universidad Tecnologica de Pereira.

**Results:**

252 patients were included, 180 received empiric therapy and 72 were FAP panel guided. The median age was 65 years (IQR 53-73), the PaO2/FiO2 ratio mean was 108 (IQR 64-130). In the group of empiric treatment, 21 (11.67%) patients presented confirmed bacterial infection. Patients on guided antimicrobial therapy presented less HAI (RR 0,54 (IC 95% 0.30-0.95) p 0.02). The median length of stay in ICU was 16 days for both groups. *Klebsiella pneumoniae* was the most frequent bacteria identified during the first episode of infection followed of *Pseudomonas aeruginosa*. Mortality on guided group was 54% Vs. 42% on empiric group (p< 0,3). Meropenem was the main antibiotic prescribed (DDD empiric 3.17 Vs. 1.8 DDD guided) followed of cefepime (DDD empiric 0.9 Vs. DDD guided 0.12). The median cost of antimicrobial treatment in the empiric group was US$530 (US$30-US$1579) per patient compared to the median cost of guided prescription that was US$292 (US$16-US$8767). When including the cost of FAP panel, the median cost per patient treatment course was US$429 (US$153-US$8904) p< 0.7.

**Conclusion:**

Implementation of a guided antimicrobial therapy using FAP panel could be useful and cost effective in COVID-19 ICU patients to reduce antimicrobial consumption and adverse outcomes related to the inappropriate use of antibiotics without significant impact on mortality or length of stay.

**Disclosures:**

**Karen M. Ordonez, MD**, AstraZeneca: Expert Testimony|Biomerieux: Expert Testimony|Farma de Colombia: Expert Testimony|MSD: Expert Testimony|Pfizer: Expert Testimony.

